# Case report of spuriously low sodium and calcium in a 36-year-old male in primary care

**DOI:** 10.11613/BM.2020.021001

**Published:** 2020-04-15

**Authors:** Seán J. Costelloe, Kelly McCarthy, Marguerite O’Connell, Mark Butler

**Affiliations:** Department of Clinical Biochemistry, Cork University Hospital, Wilton, Cork, Republic of Ireland

**Keywords:** delayed separation, spurious hyponatraemia, spurious hypocalcaemia, haemolysis, case report

## Abstract

An unseparated serum specimen for a 36-year-old male was received from primary care. The specimen arrived in the laboratory at Cork University Hospital one day after collection, as documented on the paper request card, and was promptly centrifuged. Analysis was delayed for three days due to operational constraints and serum indices were run at the same time as the biochemical analyses. Results showed a moderately haemolysed specimen with remarkably low concentrations of both sodium (119 mmol/L) and total calcium (1.15 mmol/L), with all other parameters within their appropriate reference intervals (RIs). The complete report was released electronically and both sodium and calcium results were phoned to, and acknowledged by, the requesting general practitioner (GP). Discussion between the medical scientists and clinical biochemist on duty raised the possibility that the specimen was significantly older than initially thought. Further discussion of results with the GP clarified that the documented time of collection corresponded with specimen receipt by the courier, rather than the time of phlebotomy. Thus, the specimen was 7 days old when received in the laboratory and 10 days old when analysed. This case illustrates the dangers of multiple convergent preanalytical errors. Laboratories should be mindful of the stability of analytes in unseparated blood and unusual patterns of results which might suggest a specimen is “old”, and that this may coexist with erroneous request information. Any potential adverse effects on patient care were prevented in this case by laboratory vigilance.

## Introduction

Over the past decade, laboratories have gained an increasing understanding of the importance of error in the preanalytical phase (*1*, *2*). There is a clear need to identify, monitor, and reduce preanalytical errors (PAEs), which account for the majority of all errors in the total testing process (TTP) (*3*-*5*). A plethora of PAEs can lead to reporting of erroneous results and, in turn, lead to delayed or inappropriate patient care (*6*). The authors present a preanalytical case where a primary care specimen was received, with no clinical history, or indication for testing given. The specimen had apparently been stored and then transported at temperatures unknown over the course of one day. In discussing the case, multiple PAEs are revealed, including delayed separation of a serum sample, inappropriate storage of specimens, delayed specimen transport, delayed specimen analysis, haemolysis, and misleading date and time of collection data on the request form. The authors describe how these errors coexisted and led to the generation of spurious, but apparently critically abnormal, results. The root cause PAE (delayed separation) was obscured by haemolysis and inaccurate request information. In accordance with good laboratory practice and ISO 15189:2012 standard for accreditation, laboratories should be aware of, and vigilant to, such sources of error and wherever possible have processes and mechanisms for identification of PAE at all stages of the TTP (*7*, *8*). In regions with rapid transport of specimens to the laboratory from primary care, the effects of delayed separation on calcium and sodium concentration described may not be routinely encountered, and detection systems might not be in place. It is hoped that this case will make laboratories more alert to these specific PAEs.

## Laboratory analysis

The pertinent steps in the specimen journey are summarised in Table 2. An unseparated serum gel tube (Greiner Bio-one Vacuette tube 3.5 mL CAT Serum Separator Clot Activator (cat. 454071)) was received by the Department of Clinical Biochemistry at Cork University Hospital (CUH) for a 36-year-old male, with an accompanying paper request form. The specimen was centrifuged promptly upon receipt in the laboratory. No clinical details or patient medical history were provided. Specimens were registered on the laboratory information management system (LIMS) (iLaboratory) as *per* local procedures. According to the request form, the specimen had been taken the day before receipt in the laboratory. The specimen was analysed three days later so that the apparent age of the specimen at time of analysis was four days. Details of the analytical methodologies used for each analysis are given in Table 1. Analyses described, including serum indices, were run on a Beckman Coulter AU5812 auto-analyser (Beckman Coulter, Brea, USA) and assays were performing acceptably in the period described according to internal and external quality assurance processes. The Department of Clinical Biochemistry is accredited to ISO15189:2012 by the Irish National Accreditation Board (*7*). Upon analysis, the haemolysis index (HI) was 2 (indicating free haemoglobin concentration was in the range 1.00 - 1.99 g/L), automatically blocking release of potassium, aspartate aminotransferase, and total bilirubin results, as *per* manufacturer’s cut-offs. Sodium for the patient was 119 mmol/L (reference intervals (RI): 132 - 144 mmol/L) while total calcium was 1.15 mmol/L (RI: 2.10 - 2.65 mmol/L). All results for this specimen are given in Table 1. Both sodium and total calcium results breached the departmental critical limits for phoning of abnormal and unexpected results (Table 1), based on Royal College of Pathologist (UK) guidelines for communication of unexpected and critical results (*9*).

**Table 2:** Time-line for specimen journey

**Day**	**Time**	**Apparent age of specimen (days)**	**Actual age of specimen (days)**	**Action/procedure**
1	Unknown	N.A.	0	Actual date/time of phlebotomy
7	Unknown	0	6	Apparent date/time of phlebotomy
7	Unknown	0	6	Date/time of courier pickup
8	09:27	1	7	Date/time of receipt in the laboratory
8	Unknown	1	7	Date/time of centrifugation
11	19:33	4	10	Date/time of result generation
11	19:52	4	10	Date/time of authorisation
11	19:53 - 20:30	4	10	Date/time of discussions with GP
Date and time data were retrieved from the laboratory information management system at Cork University Hospital. Only the date of phlebotomy was recorded by the general practitioner (GP) surgery. Similarly, only the date of specimen pick-up by the courier was recorded. N.A. - not applicable.				

**Table 1:** Biochemistry results reported for patient

**Analyte, unit**	**Initial results**	**Repeat results**	**Reference interval**	**Adult male phoning limit**	**Analytical method**
Sodium, mmol/L	119	143	132 - 144	< 120 or > 160	Indirect ISE
Potassium, mmol/L	S.H.	4.9	3.5 - 5.1	< 2.5 or > 6.5	Indirect ISE
Chloride, mmol/L	96	103	95 - 107	N.A.	Indirect ISE
Urea, mmol/L	6.0	6.6	2.8 - 8.4	> 30	Enzymatic (urease)
Creatinine, µmol/L	75	75	64 - 104	> 345 (or increased 1.5 in past 7 days)	Enzymatic (creatininase)
Albumin, g/L	46	48	35 - 52	N.A.	Spectrophotometric (Bromocresol green)
AST, U/L	S.H.	N.R.	6 - 42	> 630	Spectrophotometric (Modified IFCC)
ALT, U/L	34	27	0 - 45	> 675	Spectrophotometric (Modified IFCC)
ALP, U/L	76	71	48- 135	N.A.	Spectrophotometric (Modified IFCC)
GGT, U/L	51	N.R.	0 - 55	N.A.	Spectrophotometric (Carboxynitroanilide)
Total bilirubin, µmol/L	S.H.	10	2 - 20	N.A.	Spectrophotometric (Diazo)
Calcium, mmol/L	1.15	2.56	2.10 - 2.65	N.A.	Spectrophotometric (Arsenazo III)
Haemolysis Index	2	0	N.A.	N.A.	Spectrophotometric
Icteric index	0	0	N.A.	N.A.	Spectrophotometric
Lipemic index	0	0	N.A.	N.A.	Spectrophotometric
S.H. - specimen haemolysed: results that could not be reported due to the degree of haemolysis. Please note that at the time of analysis, corrected calcium was not routinely calculated or reported. ISE – ion selective electrode. N.R. - not Requested. N.A. - not applicable: where no RI or phoning limit exists for a given measurand. AST - aspartate aminotransferase. ALT – alanine transaminase. ALP - alkaline phosphatase. GGT - gamma-glutamyltransferase. IFCC - International Federation of Clinical Chemistry and Laboratory medicine.					

## Interventions and further investigations

The senior clinical biochemist discussed the low sodium and calcium results with the clinical biochemist on duty and it was agreed that the pattern of results mirrored those expected in serum where there has been a delay of several days between phlebotomy and separation (*10*, *11*). The HI was such that neither potassium, nor phosphate, could accurately be measured. Elevations of these analytes in serum are common prompts for a laboratorian to consider delayed separation. No clinical details were provided on the test request form. In discussion with the consultant clinical biochemist, the general practitioner (GP) stated that the man was on medication for rheumatoid arthritis and that biochemistry tests were for monitoring purposes. The GP was surprised by the sodium and calcium result. However, in discussion, it became clear that the specimen was older than stated on the form, and had been taken ten days previously, and collected by the courier four days previously. Phlebotomy had been performed on a Friday afternoon by the practice nurse, missing the last courier collection for that day. Since the GP surgery was not equipped with a centrifuge, the specimen was retained in the GP surgery in an unseparated state. The GP could not describe the storage conditions for the specimen between phlebotomy and courier collection, although it is suspected it was refrigerated at 4°C. The time of collection as stated on the report card was, the clinician confirmed, written by the practice nurse and corresponded to the time and date the specimens were collected by the courier. No explanation was offered as to why specimens were not picked up by the courier for 6 days, but it is assumed they were mislaid in the GP surgery during that period. An amended report was generated with the sodium and calcium results removed. Repeat phlebotomy was suggested. All repeat analyses yielded results for analytes that were within the appropriate RIs and these are shown in Table 1.

## Solution

Blood that has not been separated yields spuriously and reproducibly low concentrations for sodium after four days, depending on temperature conditions (*10*, *11*). Calcium measurements can be spuriously low where unseparated blood has been stored at room temperature for just 2 days (*11*). This was discussed with the GP and the opinion of the consultant clinical biochemist was that this was a clear case of spurious hyponatraemia and spurious hypocalcaemia resulting from delayed separation of whole blood. The GP was advised to contact the patient and organise repeat phlebotomy. The time interval between initial release of the spurious sodium and calcium results, and the concluding discussion with the GP, was less than 1 hour (Table 2), and no decisions on patient care had been made in that period. The importance of correct request details, as well as the need to transport blood specimens to the laboratory in a timely manner to ensure accurate results, was emphasised to the GP. The GP agreed to discuss within their practice that the “date and time of collection” stated on the request card should correspond with the date and time of phlebotomy. The paper request form for the biochemistry laboratory at CUH has now been amended so that the field for “Time of collection”, have been replaced with “Time of Phlebotomy” to avoid future confusion. The event was logged on the departmental incident management system.

## Discussion

The authors describe a case of apparent hyponatraemia with co-existing hypocalcaemia in a 36-year-old male presenting in primary care. Upon investigation, several convergent PAEs were at play. Manufacturer’s instructions for the serum blood tube used state that the specimen should be centrifuged within two hours. Given the logistics of specimen transport from primary care in the Cork region, this is rarely possible. All analytes for which numeric results were reported in this case have been shown in internal studies (considering intra-individual biological variability and analytical imprecision) to be acceptably stable in whole blood up to 24 hours post phlebotomy in these serum tubes. Thus, the authors suggest that this deviation from manufacturer’s guidelines with respect to time of centrifugation would not have affected any of the results described had the specimen been < 24 hours old, as appeared to be the case upon receipt. It should also be noted that studies of other commonly encountered storage conditions at CUH (e.g. serum at 4˚C for three days) have also shown acceptable analyte stability. The HI was such that neither potassium, nor phosphate, elevations of which in serum are common clues to delayed separation, could accurately be measured. Without these flags, the documented date and time of collection was taken at face value. It was not considered by the laboratory that there might be a lack of clinician understanding that the date of “collection” applied to date of phlebotomy rather than date of specimen collection by the courier. However, this highlights an important issue; the laboratory medicine community cannot assume that laboratory parlance will be understood by our colleagues in other professions. Operational constraints were such that analysis was delayed by three days following specimen receipt in the laboratory. This lead medical scientists to worry that, not only were the sodium and calcium results abnormal, unexpected, and apparently within analyte stability limits, but they related to the patient situation four days earlier which may have deteriorated in the interim. The Department of Clinical Biochemistry at CUH is the busiest laboratory service in the Republic of Ireland, processing over 12 million tests *per* year, with GP specimens accounting for some 60% of the workload. The geography of County Cork is diverse and certain regions are remote, making specimen collection and transport challenging. Where phlebotomy at a GP surgery occurs on a Friday, it is not uncommon for specimens to be three days old when received in the laboratory on the following Monday (or four days old if received on a Tuesday following a bank holiday weekend). Delayed separation of blood specimens for serum or plasma analysis can affect the stability of analytes for measurement. Identification of this common PAE requires complete and accurate data on date and time of phlebotomy as well as application of stability limits for analytes, which are appropriate for a given blood, tube type, manufacturer, mode of transport, analyser, and assay formulation. Robust laboratory processes are required to draw all these data together to accurately detect specimens unsuitable for analysis due to delayed separation. When such processes are lacking, artefactual or misleading results may be generated and reported, resulting in inappropriate and potentially harmful patient management.

## What you can do to prevent such errors?

Ensure that users of the laboratory are aware of the importance of accurate data on “time of collection”, the need for prompt centrifugation and specimen transport to the laboratory.

The laboratory should have processes in place to alert staff to potentially old specimens. For example, delta changes would have flagged both sodium and calcium results in this case. Electronic rules in LIMS or middleware systems, based on analyse stability limits, should be used where possible to reject specimens too old for analyses at the time of request generation.

Automatic or manual recognition of patterns associated with delayed separation. The patterns of results described in this case gave a clue to the root cause, although patient results were already released by that time.
